# Teams, Tools, Processes and Resources to Manage Oncologic Clinical Decision Support: Lessons Learned from City of Hope’s Multistate, Academic, and Community Oncology Enterprise

**DOI:** 10.3390/jcm14062048

**Published:** 2025-03-17

**Authors:** Linda D. Bosserman, YiHsuan Lin, Sepideh Shayani, Brian Moore, Denise Morse, Emmanuel Enwere, Vijay Trisal, Wafa Samara

**Affiliations:** 1 Department of Medical Oncology and Research Therapeutics, City of Hope, Duarte, CA 91010, USA; 2 Department of Pharmacy, City of Hope, Duarte, CA 91010, USA; ylin@coh.org (Y.L.); sshayani@coh.org (S.S.); wsamara@coh.org (W.S.); 3 Department of Physician Services, City of Hope, Duarte, CA 91010, USA; brimoore@coh.org; 4 Department of Quality, City of Hope, Duarte, CA 91010, USA; dmorse@coh.org; 5 Department of IT Pharmacy Oncology Systems, City of Hope, Duarte, CA 91010, USA; eenwere@coh.org; 6 Department of Surgery, City of Hope, Duarte, CA 91010, USA; vtrisal@coh.org

**Keywords:** clinical decision support systems, CDSS, oncology pathways, computerized physician order entry, CPOE, oncology treatment plans, EPIC, Beacon, resources, program framework, high-quality cancer care

## Abstract

**Background/Objectives:** Clinical decision support systems (CDSSs) consisting of Computerized Physician Order Entry (CPOE) and oncology pathways serve as the foundation of high-quality cancer care. However, the resources needed to develop and maintain these systems have not been characterized for oncology enterprises. **Methods:** Executive leadership appointed a medical director and clinical pharmacist to develop and lead a Pathways and Protocols Program for the City of Hope (COH) enterprise. This involved developing a program charter and governance committee and a business case for resources to support CPOE in our Epic Beacon treatment orders. Missing CPOEs for oncology treatments were identified for treatments in COH’s Elsevier ClinicalPath treatment pathways and for those few diseases not in the pathways for medical oncology and hematology. New FDA oncology drug approvals were used to estimate ongoing CPOE build needs. Time estimates for Beacon analysts to build Beacon protocols were developed from a prior CPOE catch-up project, from informal surveys of our clinical pharmacists and Beacon leads, and surveys of staff leads at two other large, multisite cancer programs using Epic. Informal surveys of oncology clinicians and pharmacists were carried out to understand the time they were using to build Beacon orders that were not in the COH system. This information was used to build a business case for additional project management and staffing to catch up on building 400 missing Beacon orders, to maintain Beacon orders as new therapies and regimens are needed, and to provide required regulatory oversight of Beacon orders. Given these standards had not been shared by others, this work was gathered into a manuscript to help others evaluate and support needed resources to manage oncology pathway programs and CPOE to improve efficiencies, safety, and quality of care for medical oncology and hematology programs. **Results:** A Pathways and Protocols program was developed with a governance committee, a program charter, and a charge for disease committees to prioritize, approve, and oversee the regulation of COH’s Beacon treatment orders. CPOE resources to catch up and maintain COH’s Beacon treatment orders were developed and shared with COH’s executive leadership. Informal surveys were completed to benchmark Beacon resources with COH and two other Beacon enterprises as well as to estimate the time used by COH clinicians to build Beacon orders for orders not in the system. **Conclusions:** The resources for managing clinical oncology pathways and CPOE for an enterprise have not previously been published. Work components identified from our work at COH are shared so that other oncology leaders might have a starting framework to evaluate their own CDSS needs for oncology pathways and CPOE.

## 1. Introduction

Clinical decision support systems (CDSSs) in oncology are now a foundational expectation for enterprise support and oversight to deliver high-quality cancer care [1,2]. The two main components of CDSS for cancer therapies are oncology clinical pathways (OCPs) and Computerized Physician Order Entry (CPOE). Yet, the framework to understand, resource, and budget for the teams, tools, and processes to implement, maintain, optimize, and learn from these CDSS components has not been defined. City of Hope uses the Epic Beacon cancer treatment orders (called protocols by Epic) for CPOE and Elsevier’s ClinicalPath cancer pathways for medical oncology, hematology, and radiation oncology decision support. COH’s executive leadership appointed a medical director to work with an informatics pharmacist to develop an enterprise-wide Pathways and Protocols program for the enterprise. They asked for a business case to understand the resources needed to update and maintain CPOE for the Beacon oncology treatments for medical oncology and hematology. The results of this work may help others organize and resource their CDSS.

### 1.1. CPOE Is Fundamental to CDSS and High-Quality Cancer Care

Recognition of the need to standardize complex cancer therapies and tailor them to individual patients dates to the Institute of Medicine’s 1999 report, “Ensuring Quality Cancer Care”. The report emphasized the importance of prescribing treatments based on patients’ specific tumors and overall health [3]. In their subsequent 2013 report, “Delivering High-Quality Cancer Care: Charting a New Course for a System in Crisis”, the Institute outlined ten goals to support the main components of their framework for delivering high-quality care. Although not explicitly mentioned, Clinical Decision Support Systems (CDSS) with clinical pathways and COPE are fundamental components for addressing their six pillars of high-quality care: shared decision-making, workforce support, evidence-based care, learning health systems, measuring and improving quality care, and expanding access and affordability [4]. More recently, in October 2023, the National Cancer Policy Forum and the American Society of Clinical Oncology (ASCO) co-hosted a workshop to evaluate progress in the ten years since the 2013 IOM report. Sadly, the workshop concluded that while progress has been made, most of the original challenges persist. Specific to CDSS, they call out the need to “Redesign the EHR to provide more useful information to support patient care; improve clinical workflow and clinician well-being; and generate evidence to support a learning health system” and the need to “Leverage digital innovation to support all aspect of cancer care” [5].

The 96% adoption rate of electronic health records (EHRs) by hospital systems in the United States can be attributed to the 2009 Health Information Technology for Economic and Clinical Health (HITECH) Act. This legislation funded health information technology and established use standards for hospitals and practitioners through Medicare’s Meaningful Use program from 2011 to 2015 [6]. As a result of this initiative, EHR adoption increased significantly, including in oncology. During this period, there was also a substantial shift in oncology services, with most cancer care transitioning from community-based practices to larger healthcare systems. This trend led to the widespread adoption of electronic health records (EHRs), with Epic (Verona, WI, USA) emerging as the dominant EHR in US healthcare, having been rated as the top overall software suite for the past 14 years in KLAS’s surveys. As of January 2024, Epic is utilized in 40% of all US hospitals, followed by Cerner at 22% and Meditech at 13% [7]. As of August 2024, Epic was used in 3000 hospitals and 67,000 clinics, including 20 of the 20 top hospitals rated by US News and World Report. Epic is the EHR used by 580,000 physicians, serving 325 million patients worldwide and 280 million in the US [8]. Epic’s comprehensive support ranges from hospital and outpatient care to telehealth, billing, collections, and integration of outside medical records. Epic offers specialized modules, developer support, and dedicated oncology steering boards to drive further advancements in oncology care [9,10,11,12,13]. What has been missing, however, is data to understand the time and staffing needed to manage, build, update, and oversee Beacon protocols and order updates for a healthcare system, as well as their integration with oncology pathways and clinical trials, engagement of staff and faculty along with outcome analytics.

CPOE has been documented to significantly decrease near-miss incidents, as well as severe and life-threatening toxicities, particularly for cancer patients [14,15]. Chemotherapy medication errors can occur at any stage of the process, including premedication, hydration, incorrect dosing, adjustments for toxicities, drug sequencing in combination treatments, infusion timing, drug dilution, scheduling, dose modification or delay criteria, and emergency medications. American Society of Clinical Oncology (ASCO) experts have established eight principles for safe EHR use in chemotherapy orders. These principles focus on ensuring accuracy, standardizing orders, automating calculations, integrating orders into workflows, providing decision support through alerts and dose maximums, allowing flexible changes as needed, and supporting efficiency, reliability, and usability. They have also stressed the importance of prioritizing safety over convenience due to the high risks of chemotherapy-related errors for vulnerable cancer patients [16,17].

The Joint Commission has identified eliminating medication errors as a primary focus in improving US health care. They supported CPOE and the Healthcare Failure Mode and Effect Analysis (HFMEA) methodology in 2000 to reduce medication errors. This approach applies engineering principles to promote reliability, safety, and quality to health care processes. A comprehensive assessment for a cancer center to use the HFMEA methodology to evaluate the risks and vulnerabilities in chemotherapy processes. After identifying 11 potential causes for four failure modes in outpatient care, CPOE for chemotherapy orders was recognized as the top priority project for risk reduction. The resulting 2008 CPOE project led to a significant decrease in chemotherapy prescription errors from 3.34% to 0.40%. This progress was made possible by reducing errors in ordering, preparing, compounding, dispensing, and administering chemotherapy. This is consistent with findings from other studies, which have shown error rates of 13% for handwritten chemotherapy orders and 5–15% error rates for paper-based chemotherapy orders are significantly reduced through standardized Computerized Physician Order Entry (CPOE) [12,15,18].

Reducing medication errors remains a significant focus of quality improvement, as described in the ongoing reviews and work by The Joint Commission and the Leapfrog Group [19,20,21]. Studies of the prevalence, factors, and interventions to reduce these errors are summarized in studies and systematic reviews. While organizations need to prioritize the building of the most used orders, it is also essential to ensure that even rarely used drugs and regimens are built to the same comprehensive organizational standards as when a drug or regimen is used infrequently, recall and experience to catch near miss errors is compromised and the risk of errors is increased [22]. This is even more important for chemotherapy orders as the therapeutic window is narrow, and the toxicities can be severe and life-threatening [23].

Also important is the standardization and regular review of built order sets. The Institute for Safe Medication Practices (ISMP) and the Centers for Medicare & Medicaid Services (CMS) have published guidelines regarding CPOE. The 2010 ISMP guidelines include standard order components and formatting, content development guidelines, and approval and maintenance recommendations. The ISMP recommends a biannual review of order sets to ensure the contents are up-to-date and relevant [24]. The Center for Medicare/Medicaid Services (CMS), through the office of the National Coordinator for Health Information Technology, has issued Safety Assurance Factors for EHR Resilience (SAFER) guidelines [25]. Each guide has recommended best practices designed to help an organization reduce EHR-related patient safety risk, optimize the safe use of EHRs, and create a ’culture of safety’. The CPOE guide has three domains (Safe Health IT, Using Health IT Safely, and Monitoring Safety) addressing 29 best practices. The two most relevant to the work discussed here are Recommended Practice 1.2, ‘Evidence-based order sets are available in the EHR for common tasks and conditions and are updated regularly’ and Recommended Practice 2.2, ‘Clinicians are engaged in implementing, reviewing and updating CDS’. The Pathways and Protocols program that was developed at COH will provide disease teams and a governance committee to oversee the biannual review of Beacon orders to meet recommendation 1.2 and their review of Beacon orders and ClinicalPath navigations meets recommendation 2.2 [25].

The Beacon module by Epic offers CPOE for complex oncology therapies for medical and hematologic oncology treatments. However, each organization is responsible for building its oncology therapy orders according to its standards, including naming, order components, formulary, coding, insurers, and other factors. NCCN provides cancer treatment regimen templates with drug dosing and scheduling based on their guidelines and published studies that can be used as a starting point for NCCN members. Epic is working to develop ways for Epic practices to share standard order templates, starting with cooperative group clinical trial orders. Currently, however, each health care system using the EPIC electronic medical record has to create their Beacon orders from clinical content provided by their multidisciplinary disease experts or consultants, with order components decided on by the organization. Standardizing these order components is an opportunity to improve safety and quality of care but a set of order components has not been established.

In a 2024 Grand Rounds presentation, clinical pharmacists reported on the initial conversion of their EHR to Epic with Beacon cancer treatment orders for Thomas Jefferson University’s Kimmel Cancer Center. They identified the need for a formal process to review their existing treatment plans and standardize the components for their Epic Beacon order builds for anticancer infusion orders. They excluded oral chemotherapy orders and research orders without standard-of-care treatments. For this project, they identified 15 cancer types and procedures (BMT, CAR-T, breast, CNS, GI, GU, GYN, head and neck, thyroid, leukemia, lung, lymphoma, melanoma, multiple myeloma, and sarcoma) which had 1036 orders to be reviewed, standardized, and built. They specified components for their new Beacon orders, including the drugs, supportive care medications, required laboratory orders, and other monitoring recommendations to ensure safe and effective care based on the FDA package inserts. Of the first 375 orders reviewed for five cancer types, 311 orders (83%) needed updating, with an average of 1.7–3.07 interventions per order [26]. The US Oncology group also identified the need to standardize their treatment order sets after reviewing the 511 cancer treatment orders in their iKnowMed EHR. They found that 51 treatments could be removed or consolidated and that edits were needed in the remaining 460 treatment orders. The identified needed edits needed to standardize, update, or correct drug dosing, drug cycles, treatment references, and naming information [27].

### 1.2. Oncology Clinical Pathways Are the Other Key to CDSS and High-Quality Cancer Care

The second component of CDSS, oncology clinical pathways has also been shown to improve efficiencies, value, and quality of care by guiding busy clinicians from multidisciplinary experts and synthesizing complex evidence-based science [28,29,30,31]. The City of Hope uses Elsevier’s ClinicalPath oncology pathways based on their participation with national experts in leadership, development, and updates to Elsevier’s ClinicalPath Oncology pathways for medical oncology, hematology, and radiation oncology. City of Hope faculty serve on most Elsevier ClinicalPath disease committees and as co-chairs for several. City of Hope clinical trials are integrated from our OnCore system into the specific ClinicalPath pathways, so COH clinicians first view trials followed by standard of care options for their patient’s disease, stage, and biomarkers. The clinical trials are presented with the principal investigator and the sites where they are open across the enterprise. A click to select a trial notifies the clinical trial team. If a trial is not selected, the reason is noted and mineable, and the standard of care therapies are then presented.

**Figure 1:**
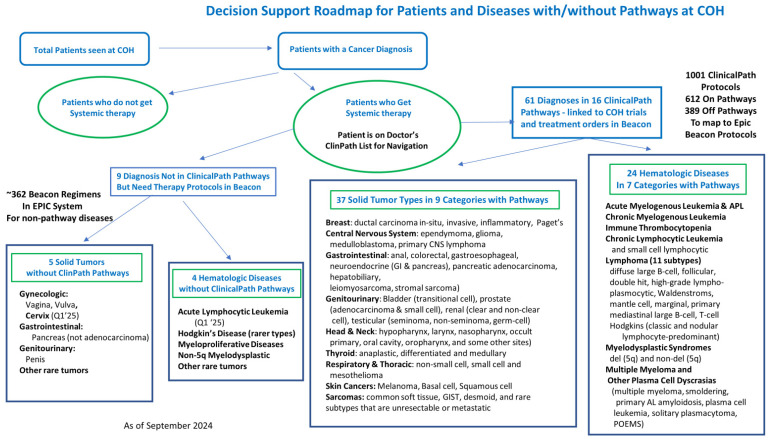
Decision support roadmap for patients and diseases with and without ClinicalPath pathways at COH.

City of Hope’s ClinicalPath pathways are the foundation of a four-tiered pyramid of treatment decision support that includes the Clinical Path pathways with clinical trials, formal enterprise-wide tumor boards, expert faculty consultations, and complex oncology case conferences, as previously described [32]. The ClinicalPath pathways guide initial and subsequent lines of therapy for 61 cancer types. The pathways cover 37 solid tumor types within nine categories—breast, CNS, GI, GU, GYN, head and neck, thyroid, respiratory and thoracic, skin cancers, and sarcomas—and 24 hematologic disease types within seven categories—AML and APL, CML, CLL, ITP, lymphomas, MDS, multiple myeloma and plasma cell dyscrasias. Pathways for cervical cancer and ALL will be available in 2025. The ClinicalPath system provides pathway guidance for over 99% of all cancer patient presentations across our enterprise, as shown in Figure 1.

### 1.3. COH’s Insights from Earlier Work with Oncology Pathways and CPOE

The expansion of City of Hope’s care from an initial academic site in Duarte, CA, to multiple CA community network sites led to the adoption of clinical decision support (CDS) with ClinicalPath pathways (formerly VIA Oncology) for medical oncology and hematology in 2017, and for radiation oncology in 2018. The City of Hope (COH) converted all California sites to the Epic EHR, encompassing the Willow Pharmacy application for medication management and the Beacon Oncology application for Computerized Physician Order Entry (CPOE) for cancer therapies and other EHR functionalities in December 2017. An informatics pharmacist led the pathway program and worked with the medical director of value-based care to oversee prioritization and build of Beacon orders needed for the ClinicalPath pathways, as well as work to develop informative analytics in the COH tableau system. This was followed by stepwise integrations of staging and Beacon treatment orders in Epic with the ClinicalPath navigation system for both standard-of-care therapies and, in May of 2023, for COH clinical trials. COH’s expansion from 2022 to 2024 added a second academic site, Lennar in Irvine, CA, to the long-established Duarte, CA, academic site. It expanded the California network to 32 sites and, in February of 2022, added the former Cancer Treatment Centers of America sites in Chicago, Illinois, Atlanta, Georgia, and Phoenix, Arizona. These sites, now called COH-CAP sites (for Chicago, Atlanta, and Phoenix) added nine inpatient and seven outpatient sites across the three states. By October 2023, the CAP sites went live on COH’s California Epic electronic health record. In preparation for transitioning the CAP sites onto EPIC, their treatment protocols and antiemetics were reviewed, and it became clear that to provide a uniform standard of care across the enterprise, a Pathways and Protocols program was needed.

Prior to the development of this multistate approach, modifications and additions to Beacon orders were made from submitted requests by clinicians and Pharmacists to COH’s CO”sCOH IT team via a trackable ticketing system or by the informatics pharmacist and medical oncologist collaborating with disease teams to identify needed Beacon orders. These two individuals also coordinated two earlier CA-wide projects to improve COH’s Epic Beacon protocol builds and antiemetic standardization using existing and some additional budgeted resources from the precision oncology team. A review of these project highlights informed some aspects of the current work to update antiemetics across the enterprise and to document the resources needed to catch up on unbuilt orders as well as maintain and oversee regulatory work to maintain the Beacon treatment orders going forward.

Initial work for our EPIC go live in 2017 had standardized antiemetics into 26 oral and 26 IV dominant order sets to address the various highly emetogenic chemotherapy (HEC), moderate emetogenic chemotherapy (MEC), low emetogenic chemotherapy (LEC) and minimal emetogenic chemotherapy (MIN) risks in treatment protocols. In 2020 the first of the two CPOE projects involved updating our antiemetic regimen standards based on the updated National Comprehensive Cancer Network (NCCN) guidelines [33]. These included adding an option for olanzapine to highly emetogenic chemotherapy (HEC) regimens, reclassifying antiemetic levels to highly emetogenic chemotherapy (HEC) from moderate emetogenic chemotherapy (MEC) for carboplatin regimens with AUC > 4, and changing to more optional use of lorazepam with dosing guidelines for older patients to reduce fall risks. Despite this work, a search in 2022 showed over 157 antiemetic regimens in our Epic system. These had been built at the request of individual doctors working with various Beacon builders without the oversight of a formal Pathways and Protocols program which supported the current move to an enterprise-wide governance structure to oversee standardization.

In 2020, a second CPOE project was identified after recognizing that oral chemotherapies needed to be built as Beacon protocols in Epic in addition to those for IV, IM, and other treatment orders. The need to standardize the growing number of oral chemotherapy treatment orders was supported by studies as a vital component in reducing chemotherapy order errors [23]. In total, 106 oral chemotherapy treatment orders were identified as needing to be built into Epic Beacon protocols to coordinate with ClinicalPath pathway therapies. Funding was obtained from the precision oncology team for additional staff to build oral Beacon protocols as well as 34 other regimens requested by clinicians for a goal of 140 protocols. Over 6 months from March 2022 through September 2022, academic disease leads and community oncologists volunteered to provide the clinical content. The disease leads and the PharmD and nursing disease specialists worked with the VBD and informatics pharmacist leads to edit and validate the final Beacon protocol builds before sending them to the P&T committee for approval and activation in Epic. Over the six months, USD 150,000.00 was used to fund a project manager and two Beacon analysts to build treatment orders. The two analysts spent 1248 h building and editing 133 new Beacon protocols. In total, 84 oral protocols were completed and moved into production (breast (25), lung (6), myeloma (17), skin cancer (2), adrenocortical cancer (1), chronic graft vs. host disease, (cGVHD) (2), chronic lymphocytic leukemia (CLL) (12), chronic myeloid leukemia (CML) (5), colorectal (1), idiopathic thrombocytopenic purpura (ITP) (4), melanoma (3), myeloproliferative diseases (2), myelofibrosis (2) and prostate cancer (2)), 25 other protocols were moved into production, and 13 (10 oral) were built but awaiting P&T approval over the six months of the project. Analysts documented an average of 9.38 h per finalized protocol. Of the original 106 oral regimens identified, 10 were deemed no longer necessary, and 10 were awaiting clinical content when the project ended. This established the COH’s experience of 9.38 h of Beacon analyst-builder time per protocol as an average for business case development.

### 1.4. Current Need for Enterprise-Wide Pathways and Protocols Program and Business Case for CPOE

The literature emphasizes the importance of CPOE as part of Clinical Decision Support Systems (CDSSs) in oncology to minimize potentially severe or life-threatening toxicities and to optimize outcomes for evidence-based cancer therapies personalized to each patient [23]. As a significant component of CDSS, oncology clinical pathways have also been shown to improve efficiencies, value, and quality of care by guiding busy clinicians from multidisciplinary experts and synthesizing complex evidence-based science [26,27,28,29]. While the literature has discussed the importance of CDSS and Dr. Weese and colleagues published their work across their multiple sites to incorporate Beacon protocols and clinical trials with the Aurora Cancer Center ClinicalPath pathways in their Epic system in preparing for value-based care [34], a framework to understand the resources and organizational processes for CDSS for oncology programs has not been developed. Thus, we share the work at COH in the hopes of helping others faced with these challenges.

Over the last 5 years, City of Hope’s multistate expansion and conversion of all sites to the EPIC electronic health record necessitated the establishment of a more formal program to oversee the thousands of Beacon treatment orders, provide periodic updates to order components including antiemetic regimens, establish standardized components for Beacon orders, set priorities for building missing and new therapies, develop a formal regulatory review of Beacon treatment orders, manage clinical trial integrations, updates and utilization of the clinical pathways, and oversee analytics for the ClinicalPath oncology pathway program. Methods used and results are shared so that others can build their own frameworks to support this work at other institutions.

## 2. Materials and Methods

A medical director was appointed by COH’s chief clinical officer to lead an enterprise-wide Pathways and Protocols Program. An experienced clinical pharmacist was hired, and a part-time program manager was assigned. This core team worked with COH’s pharmacy and informatics leaders to develop an Enterprise Pathways and Protocol Program to oversee the Elsevier ClinicalPath (Elsevier, Amsterdam, The Netherlands) pathways which are updated quarterly and used by medical oncology, hematology, and radiation oncology and cancer therapy orders written in COH’s Epic Beacon module (Epic, Verona, WI with their quarterly updates implemented biannually at City of Hope) orders for medical oncology and hematology diseases with and without pathways in ClinicalPath. This core team met weekly for a year and continues to meet biweekly to lead the program. Project management principles were used to develop program goals, a proposed charter, and a governance structure. An enterprise multidisciplinary governance committee first convened in November of 2024 and meets quarterly to provide input and oversight for the program.Regarding the ClinicalPath pathways, the core team met with ClinicalPath leads at COH and Elsevier, with COH’s analytic and quality staff, with disease leads appointed by the chairs of medical oncology and hematology, regional leads, departments, and individual clinicians as needed to optimize efficiencies with using the pathway system within Epic toward COH’s goal of having at least 80% of all treatment decisions navigated in the ClinicalPath system. Understanding the current and optimal state of Beacon treatment orders was needed to improve ordering efficiencies, safety, and quality care, as well as their integration with ClinicalPath pathways. COH developed a list of treatment regimens from ClinicalPath pathways and matched them to regimens in our Epic Beacon orders for medical oncology and hematology and to the Aria treatment orders for radiation treatments. Tableau reporting for every COH site and clinician was developed with data from our data warehouse, the Aria radiation therapy system, and weekly ClinicalPath navigation reports. Weekly dashboard reports were developed and, starting in August of 2024, are sent to clinicians and regional and department leaders, showing the numbers of new therapies ordered against the number navigated in the ClinicalPath system or Aria system. These dashboards have details available for therapies and navigations by clinician, patient, disease type, site, region, and state for medical oncology, hematology, and radiation oncology. Work is ongoing to adjust reports for clinicians who join or leave COH as well as ensuring every oncology treatment plan ordered in Beacon is tracked for having a pathway or not in the ClinicalPath pathways. ClinicalPath has pathways for the majority of all therapies provided at City of Hope. When choosing a treatment plan in Epic Beacon using the ClinicalPath pathways, staging data are pulled from Epic into the pathway system, and clinicians navigate to the treatment plans where the clinical trials are shown followed by the standards of care by efficacy, toxicity, and cost as well as for some patient-specific needs. The therapy choice is then reportable by treatment category as a clinical trial, a standard of care, or an off-pathway choice. Monthly meetings with regional operational leads and executive leaders are held to review ClinicalPath navigations. Incentive payments for navigating treatment choices in the ClinicalPath system, but not based on the choice of treatment, are made by leadership and vary by the site of care. Incentive payments have increased navigation work by clinicians as it requires additional time for data capture. Many disease super-specialists or doctors who focus on only one or two cancers know current clinical trial options and standards for treatment choices; they do not rely on the pathway system to guide their treatment choices. Their treatment choice navigation data, however, is important to the institution to understand clinical and financial outcomes by disease, stage, and biomarker and for various value-based payer contracts. All clinicians are currently expected to navigate their treatment choices in the ClinicalPath system. In reality, the navigations vary across sites from 30 to 100% with more community sites where oncologists and hematologists see patients with many different cancers daily and use the pathway system routinely in confirming their treatment decisions.

Oversight of CPOE as Beacon treatment plans was the other focus of the core team starting in late 2023 and ongoing. The core team pharmacist surveyed the protocols built in our Epic Beacon module in 2023 and compared them to those in the ClinicalPath pathways and those requested by pharmacists and clinicians for new drugs or regimens for non-pathway diseases. The team reviewed the pace of new drug and treatment orders needed over the past 2 years and estimated ongoing needs for 15–18 new Beacon treatment orders monthly given the continued pace of new drug development and expansion of use for drugs originally approved for other diseases. To check whether the number of missing Beacon orders was valid, the clinical pharmacist queried our Epic system to see how many Beacon orders were built ‘on the fly’ by physicians or pharmacists over the 2-year period from 2021 to 2023 because they were not built into the system. These data were primarily from California sites as the CAP sites had only been live on Epic for 3 months of the study. These data confirmed that many needed regimens were being built individually. Given the immediate focus for such builds on getting patients treated, it was noted that those orders focused on drug therapies and did not always contain all the other order components to coordinate downstream work such as education visits, preauthorization, emergency medications, and standardized antiemetic regimens. This led the group to develop and obtain approval across the organization for a standardized set of Beacon order components that would be required for analysts to build into all new and updated Beacon orders in our system.

After gathering the data on new and ongoing Beacon treatment orders needing to be built, the group was charged with building a business case for the additional resources needed to address the work to catch up and maintain the Beacon orders. Maintaining the orders included recognizing the regulatory requirements to formally review and update current and future Beacon orders at least every 2 years. To estimate the building resources for catch-up and maintenance, the group used data from the 2022 oral chemotherapy build project, estimates from our Epic Beacon leaders based on current capacity and output, and an informal survey of leadership known to the core team from two other large, multisite oncology programs who use Epic Beacon for treatment orders. Given the time limits for the needed business case, there was no time to carry out a formal benchmarking study of resources used across all large Epic oncology organizations.

With the addition of the CAP sites in three other states, clinical pharmacists across the enterprise reviewed antiemetics. Palonosetron was adopted as the preferred IV 5-HT_3_RA instead of COH’s previous ondansetron choice. The antiemetic regimens were updated, and standards were developed for 47 unique antiemetic regimens, 23 IV dominant and 24 oral dominants to cover the HEC, MEC, LEC, and MIN antiemetic risks in protocols given on different days and with integrated hypersensitivity medications. Given different preferences for all oral or a combination of IV d1 and oral pre/post meds as needed, the COH clinicians have demanded having antiemetic regimens built two ways for every Beacon protocol to rapidly choose their preference, generally by site or region or payer requirement. Additional resources were identified as part of the CPOE catch-up and maintenance project to obtain the new antiemetic regimens built in Epic and available to be used in all new Beacon treatment orders and then edited into all current Beacon orders as they come up for regulatory review or as resources allow. The antiemetic regimen standards developed by the enterprise pharmacy team were subsequently agreed to by the medical oncology, hematology, and nursing departments and approved by the Pathways and Protocols governance committee.

After the team’s initial work indicated significant additional resources would be needed with significant costs to build new antiemetic regimens, catch up and maintain and oversee edits and regulatory reviews of Beacon treatment orders, the core team engaged in informal surveys of clinical pharmacists and oncology clinicians to understand the number of hours being used by their clinicians to build Beacon orders that were not built or built correctly in the system. The program leads called and sent email surveys asking regional leaders of medical oncology, hematology, and pharmacy to survey their staff and provide an estimate of the number of hours per clinic day that their PharmDs and clinicians were spending. The data were surprisingly consistent and indicated an enormous burden of wasted hours by clinicians and PharmDs that could be redirected to seeing patients, which would produce additional revenue. A general estimate that every 4 h saved by a clinician could, very conservatively, result in seeing one more new patient would allow each regional lead to estimate the revenue increase per site. This would be in keeping with one of COH’s main goals of seeing more new patients with shorter wait times. The hours wasted were provided in the business case, but the final calculations of revenue benefits per site for seeing more new patients were left to senior leadership, who could use their information to understand how the additional investment in Beacon order build resources could provide an ROI that varies by site, by region, and by state.

## 3. Results

### 3.1. Enterprise Clinical Pathways and Protocols Program Development

- Program leadership: Our executive sponsor is our Chief Clinical Officer (VT). He appointed a part-time medical director (LB) in September 2023 who co-leads the program with the program’s clinical pharmacist (YL) hired in December 2023. They work with a part-time program manager (BM) as a core team. They actively interact with the clinical pharmacy, IT, ClinicalPath, and Beacon leadership, department chairs, faculty, nursing, and operations leadership.
- The program charter codified the membership, meetings, objectives, reporting structure, scope of work, and deliverables. The core team developed the charter, which was reviewed and approved by the medical oncology, hematology, and radiation oncology departments and the governance committee.
- A governance committee was formed with multidisciplinary stakeholders across the enterprise. They meet quarterly and oversee pathway and Beacon protocol policies, standardize Beacon order components, update antiemetic guidelines, ensure regulatory compliance, support resources to meet the program goals, and review analytics to document our high-quality care.
- Disease teams were formed for each disease or disease subtype at the preference of the disease lead and department chairs. The teams are led by an academic faculty member appointed by the department chair. They consist of a clinical pharmacy and nursing disease expert, as well as any interested faculty across the enterprise who wants to attend. Attendance from physicians, APP, nursing, and pharmacy representatives from the CA community, CAP sites, and the Duarte and Irvine academic sites is welcomed. Epic Beacon and IT staff are encouraged to attend as well. COH has disease teams for pathway and non-pathway diseases as follows:
  ▪ Solid Tumors:
    - Breast, GU, GI by subtype, gynecology, head and neck, lung, melanoma and skin, brain, sarcoma and soft tissue, thyroid.
  ▪ Hematology:
    - Lymphoma by subtype, ITP, myelodysplasia/myeloproliferative, myeloma and plasma cell diseases, CML, AML, ALL, CAR-T, BMT.
    - Although BMT and CAR-T therapies are recommended as appropriate in the ClinicalPath pathways, we have separate disease committees at COH to oversee the Beacon protocol builds and other components of those therapies.

The disease teams have a defined agenda for the Pathways and Protocols program meetings. They meet monthly unless less frequent meetings can address the needs of their disease. The disease team meetings address the following:

- Overseeing Beacon protocol builds, identifying and prioritizing builds;
- Providing content for regimen builds, validating builds when done, and sending to COH’s P&T committee for final approval before going live in Epic;
- Reviewing ClinicalPath updates and any pathway issues;
- Reviewing COH clinical trials in the ClinicalPath system;
- Regulatory review of Beacon orders biannually;
- Reviewing any analytics of interest on pathway choices and clinical trial accruals.

### 3.2. Current State Beacon Treatment Order Gap Analyses and Time Impact Surveys

- Gap analyses and prioritizations for missing protocols were performed to determine the number of Beacon orders missing from our Epic system. City of Hope’s Epic system had 1100 Beacon protocols built. Built protocols were matched against the 612 ClinicalPath pathway regimens for the 61 diseases in the ClinicalPath system as of April 2024. The addition of AML pathways in September 2024 added 72 more protocols to be mapped to our Beacon system. COH’s remaining orders are associated with COH’s other standard-of-care therapies and procedures that are not covered or detailed in the pathway system (primarily BMT, Car-T, TIL, cervical, ALL, and Myeloproliferative diseases).

The 326 regimens identified as missing from the current Beacon protocols were divided into their respective disease pathways. The disease leads and pharmacists were then surveyed to determine the usage frequency to prioritize getting them built. The prioritization criteria were divided into four categories based on order frequencies: priority 1: once every 1–4 weeks; priority 2: 1–3 months; priority 3: 4–12 months; or priority 4: rare or not currently used. This resulted in 36 priority 1, 75 priority 2, 89 priority 3 Beacon protocols, and 126 noted to be used rarely or not needed. Caution was advised as business principles prioritize treatments used most frequently. Data, however, suggest that even rarely used protocols should be built as part of CPOE. Clinicians building regimens used infrequently may not recall specific dosing alterations, supportive care, and other needs of rarely used therapies. The results of the prioritization for the 326 missing protocols are shown in Table 1.

**Figure 2:**
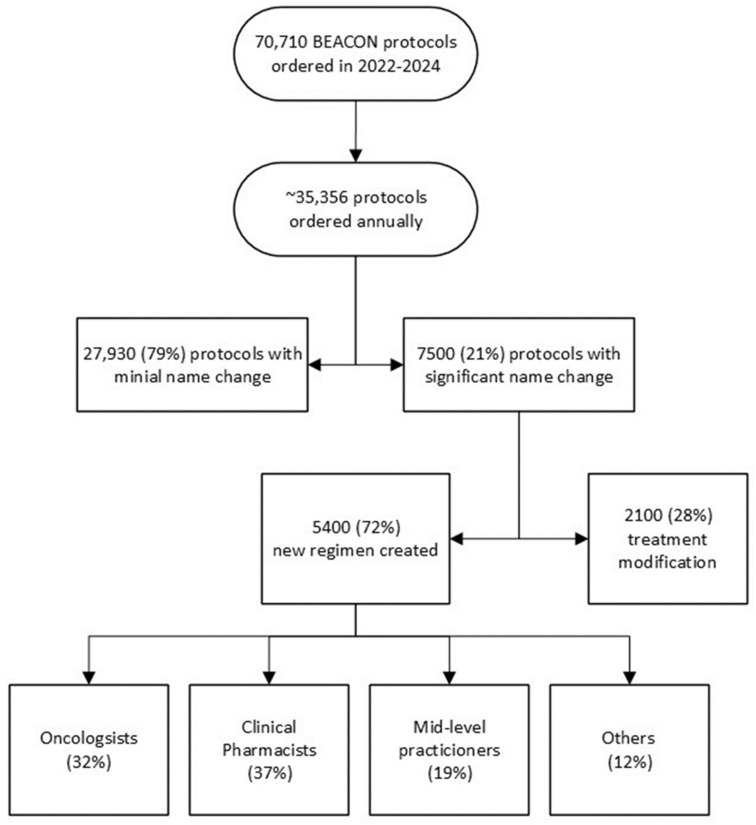
Beacon Protocols built on the fly over two years by specialists based on Epic data.

- Gap analysis of Beacon orders built on the fly over two years was performed to understand the impacts of unbuilt protocols. The Epic system showed that over 35,500 protocols were ordered annually, with 7500 being built or modified by clinicians or pharmacists. In total, 5400 Beacon protocols were created, while 2100 were made by modifying existing protocols to create the needed treatment. The number and staff who built the missing protocols are shown in Figure 2. The 7500 built or modified protocols represent 21% of all Beacon protocols ordered. Many protocols were built several times by different staff who needed to get their patients treated. We heard from one pharmacist who built the same myeloma regimen ten times over a few months for ten different patients.

- Three informal surveys were conducted to estimate the time spent by staff in building Beacon protocols ad hoc. While the findings varied, all three studies revealed a significant daily time commitment per clinic day from clinicians, clinical pharmacists, advanced practice providers (APPs), nurses (RNs), and others. The potential time savings, by having Beacon orders built in Epic, could be redirected towards seeing a significantly higher number of new or follow-up patients and providing more comprehensive care to those patients. For instance, the CA and CAP community site survey revealed that 250 h per month were being used by physicians to build Beacon orders. This could translate into 63 more new patients being seen monthly across those sites, assuming that for every 4 h saved, at least one new patient could be seen. This revelation carries significant implications for accelerating access to high-quality cancer care, a top priority for our enterprise.

○ The first study estimated the physician’s time to build the Beacon orders. It is based on the Epic order study discussed above. Given that 7500 annual ad hoc Beacon protocols were built and based on the time experienced builders take to create or modify Beacon orders, estimates of the time used were made. Treatment plan modifications included the time to change orders in Beacon and to review and ensure the accuracy of the changes. Experienced Beacon order builders estimate the time needed to be 5 to 40 min, depending on the complexity of the modifications or new builds. When the combined MD, APP, RN, and PharmD times were evaluated, it was estimated that at least 128 h per month were being wasted on these tasks that could be centralized. These data were primarily from the California network and academic sites, containing only 6 months of CAP site data as they transitioned to Epic in October 2023. Table 2 shows how the estimated wasted time was calculated.
○ The second survey of medical oncologists’ and hematologists’ time estimates to build Beacon orders was based on emails and discussions with the regional site leads for the California community network and CAP sites. They were asked to survey their doctors about the time their doctors were using each clinic day to build or modify orders that were not available. Based on the number of doctors and time spent at each site, monthly totals were calculated for wasted time per MD. Making the conservative assumption that for every 4 h freed from creating orders, a doctor could see at least one new patient, the new patient potential for all the doctors at each community site was calculated. This conservative estimate shows that at least 63 more new patients could be seen monthly at the network sites across four states to serve patients better. Results are shown in Table 3.
○ The third study aimed to report the time clinical pharmacists spent building or editing Beacon orders that were not in our system. The clinical pharmacist program lead (YL) spoke with the clinical pharmacists at each site to gather their daily hours spent editing unbuilt Beacon orders. These pharmacists worked at the Duarte and Lennear outpatient clinics as well as the three CAP sites. This analysis revealed significant daily, weekly, and monthly use of our pharmacists’ time, conservatively estimated at 40 h per day across the enterprise, which could be better utilized to serve patients and the organization. Results are shown in Table 4.

The results of these three studies confirm the significant amounts of clinician and clinical pharmacist time being used to build or modify Beacon protocols that are not in our Epic system. In addition, it was noted that many of these missing orders were being built multiple times by different people at different sites. Beacon orders built for individual patients are not available for others to use. Orders built on the fly have also been found to focus on the treatment details, and many do not include all of the standard order components that have been agreed to for efficiency and safety. Each survey, whether from the numbers of regimens built per year ad hoc or the physician or clinical pharmacists surveys, supports the argument that building the backlog of Beacon orders and keeping new Beacon orders up to date would free time across the enterprise to provide more care to current and new patients. Although the estimated additional revenue per new patient will differ for every organization, the costs of catching up and maintaining Beacon orders can be expected to be more than offset by additional patient revenues leading to a positive return on investment.

### 3.3. Evaluating the Time and Staffing Required to Build, Update, and Maintain Standardized Beacon Protocols

- Resource analysis has considered personnel needed for program management, leadership, clinical coordination, and technical support to maintain the EHR workload, catch up on current needs, and plan for future growth. Program leadership includes an MD medical director leading the pathway and protocol program and an informal pharmacist with clinical oncology knowledge coordinating interdisciplinary teamwork among specialties to ensure program development fits COH’s practice and standards. To recover the current deficit and maintain program growth, sufficient technical support is required to build Beacon protocols for on- and off-pathway regimens.
- The core team worked with the IT and Epic Beacon staff to understand the time it takes to build Beacon protocols with the standardized components for our Epic system. They reviewed data from the 2020 project to build 106 oral regimens over 6 months to meet QOPI and other oral CPOE standards, as has been discussed [16,17,25,35]. The medical director and an informatics pharmacist collected the clinical content from faculty disease leads, community oncologists, and clinical pharmacists, then worked with the budgeted Beacon analyst builders. They were able to complete 94 protocol builds (84 moved into production and 10 pending Pharmacy and Therapeutics (P&T) approval) plus 25 other protocols using an average of 9.38 h of Beacon analyst time per protocol.
- From the time impact survey of physicians and clinical pharmacists discussed previously, expert clinical pharmacists noted that even modifying Beacon orders and reviewing their accuracy can take 5–40 min, depending on the complexity of the protocol. For new protocol builds, the time to build consists of the time to gather the clinical content for each applicable component of the order, the time to build the protocol in the Epic system, the time for MD, PharmD, and RN review and validation of the Epic build, then time for any edits and re-review for validation before a final protocol order is sent to COH’s monthly Pharmacy and Therapeutics (P&T) committee for final approval. After that, an approved protocol can be activated across all of our Epic sites. An alternative methodology is for an experienced oncology pharmacist to be trained and certified as an Epic builder. They can then gather the clinical content and build the initial Epic order with all its components to avoid any back-and-forth between the clinical content provider and the Beacon analyst builder. Oncology pharmacists are the most experienced in the many nuances and details needed to build a complete oncology order protocol, but few have Epic Beacon builder certification. Thus, most organizations have clinical pharmacists or clinicians provide the content and then work with an Epic Beacon analyst builder until they are satisfied with the Beacon protocol. A disease team of an MD, RN, and PharmD then reviews the final order for any edits and their approval so it can be sent to the P&T committee for final approval and movement into the Epic system.
- The core team estimated the additional staffing needed to catch up and maintain our Beacon protocols. This was calculated to catch up 400 unbuilt protocols (326 prioritized from the ClinicalPath pathways and 71 requests for updates from clinicians to IT) and to provide the biannual protocol review for the current 1100 Beacon protocols. New protocol builds are estimated to require an average of 4 h of PharmD time, ranging from 2 to 10 h. Updates to current protocols are estimated to take 1.5 h of PharmD time, and protocol review and standardization, including the updated new antiemetic regimens, is estimated to take 2 h per protocol. Thus, the catch-up work would require 3907 h of PharmD time: (400 protocols × 4 h) + (71 protocols × 1.5 h) + (1100 protocols × 2 h), which would require two full-time PharmDs. For the Maintenance of Beacon protocols, we estimated 220 new protocols annually take an average of 4 h of PharmD time (with a range of 2–10 h), 200 clinician requests for updates or modifications annually take an average of 1.5 h of PharmD time and the ongoing biannual review of half of what will be 1500 Beacon protocols means 750 protocols need to be reviewed annually at 2 h of PharmD time each. Thus, for the maintenance of Beacon protocols, it will take 2750 h of PharmD time: (225 × 4) + (200 × 1.5) + (750 × 2), which would require 1.5 full-time PharmDs. The results are shown in Table 5.

**Table 5:** Time commitment per protocol for COH PharmDs and Epic Beacon builders.

Task	# Protocols	Pharmacist Time per Protocol	Total Time PharmD	Beacon Builder per Protocol	Total Time Beacon Builder
New Protocol Build (most complex treatment plans)	400 Catch Up Builds 220 Annual New Builds	4 h (range 2–10 h) 4 h (range 2–10 hrs)	1600 h 880 h	6.5 h 6.5 h	2600 h 1430 h
Protocol Updates based on requests	200 annually	1.5 h	300 h	4.0 h	800 h
Biannual Protocol Review and Antiemetic Updates	750 (based on 1500 total)	2 h	1500 h	2.0 h	1500 h

h and hrs = hours. # = number of protocols.

Going through a similar process for the Beacon analyst builders and Willow analyst builders along with the project manager, an overall recommendation was made for the added resources needed to catch up and maintain our Beacon protocols. These recommendations are shown in Table 6. The total budget for the catch-up work was estimated to be USD 1.9 million over 14 months. The total budget for the annual maintenance work was estimated to be USD 1.1 million.

A benchmarking study was conducted on staffing to build and maintain Beacon protocols at two large cancer organizations, and it was compared to COH’s current and proposed staffing. Two COH staff who had recently transitioned from two large, multi-state, multi-site Epic Beacon using cancer programs gathered information about the number of Beacon protocols and the resources used to build and maintain them. The results, comparing the current and proposed additional resources for Beacon protocols at City of Hope, are shown in Table 7. The benchmarking study supports the need for six additional staff to catch up on protocol builds over 14 months and then maintain COH’s Beacon orders going forward.

### 3.4. Establishing Standard Components for Beacon Protocols for the City of Hope Enterprise

Standardizing the components of our Beacon protocols ensures the order sets address the many functions needed for safe and effective patient care. A set of Beacon order components was identified, then reviewed and edited by the governance team and the departments of medical oncology, hematology, clinical pharmacy, and nursing before being finalized. They are to be considered and added as appropriate to every enterprise Beacon protocol. The components are shown in Table 8.

In addition to Beacon protocol order components, protocol formation should be standardized. The protocol should have a standardized naming convention, including commonly known treatment acronyms, individual drug names, and disease sites. The protocol description should include regimen details and emetogenicity level. The protocol should also include reference hyperlinks for the landmark trials of the treatment regimen, as shown in Table 9.

### 3.5. Recommendations for Updated Enterprise Antiemetic Standards for Oral and IV Dominant Regimens

- The clinical pharmacy team recommended that the following medications be standardized for the antiemetic regimens to meet HEC, MEC, LEC, and MIN needs in our Beacon protocols consistent with the latest NCCN guidelines and site and disease team needs. Table 10 shows the recommended drugs for the oral and IV dominant antiemetic protocols by emetogenic risk category.

- The antiemetic doses are then further adjusted when NK1 RA medications need to be deleted, or steroids adjusted or when partnered with one of the four chemotherapy regimens where steroid dosing needs to be coordinated with hypersensitivity and emetogenic prevention. Table 11 shows the different modifications of the antiemetic drug dosing to be built.

- These combinations resulted in 24 standardized regimens for antiemetics that can be added to Beacon protocols. These 24 regimens will be built in two ways: 24 as oral dominant and 23 as oral plus day of therapy IV regimens to meet different payer and care needs. These regimens were developed with enterprise pharmacy input and then reviewed and approved by medical oncology, hematology, and nursing departments. Building these pre- and post-medication protocols will be prioritized for addition to all new Beacon protocols. They will also be added to the current Beacon protocols as they are reviewed biannually or earlier, pending resource availability.

## 4. Discussion

Benchmarking and reporting on high-quality cancer care delivery is increasingly important to a healthcare organization’s reputation and success. The increasing complexity of cancer therapies tied to disease subtypes, staging, molecular and other biomarkers, clinical features, patient needs, and sequencing has led to the widespread adoption of EHRs for documentation and provision of CDSS with CPOE and oncology clinical pathways. For oncology organizations, CPOE focuses on complex cancer treatment orders. For Epic EHR organizations, oncology CPOE is carried out through the Beacon treatment protocols; yet, standards for the resources to manage, build, and oversee CPOE and clinical pathway programs for an enterprise have not been developed. Executive leaders need business cases to justify resources so the work carried out from 2023 to 2024 at City of Hope is shared so that others can use the relevant components to support the work at their institutions.

Clinical pathways can be provided as a component of CDSS using custom or third-party clinical pathway tools. These tools are ideally integrated with Epic Beacon treatment orders to prompt and guide clinical trials and standard-of-care treatment choices. Once a treatment is chosen in the pathway system, it can be linked to the Beacon protocol for efficient ordering. The ClinicalPath pathways for medical oncology, hematology, and radiation oncology meet ASCO’s established high-quality cancer care pathway criteria, as do the other nationally available McKesson Value Pathways powered by NCCN [36]. Those pathway systems have been shown to optimize costs and outcomes by the Pittsburg Cancer program, the US Oncology group, and the City of Hope using Beacon treatment orders and the ClinicalPath pathway program [34,37,38,39,40,41]. Pathway tools facilitate oncology enterprises’ prompting, delivery, and measurement of high-quality care delivery personalized for each patient based on evidence-based guidelines and their faculty’s expert oversight. The costs of such systems are proprietary, and an analysis of the costs and benefits of participating in national pathway programs that leverage central management and national faculty expertise compared to organizational pathways has not been published. Thus, benchmarking data are not publicly available for pathway programs as a component of CDSS. A framework to understand the resources to build and maintain the depth and breadth of oncology clinical pathways through participation with national groups vs. institutions or enterprises who build, update, and maintain their own pathways is beyond the scope of this project but of interest to oncology organizations working to optimize value-based care and understand the cost of having comprehensive oncology pathways for their clinicians.

For CPOE, however, standards, even for the number of protocols needed and the cost to build, update, and manage them within the Epic electronic record can be shared but have not been published. We present our work components to serve as a framework for others to evaluate their oncology treatment order needs with associated costs and resources to update, maintain, and oversee the building and regulation of Epic Beacon treatment orders. We present our work to understand the number of standard-of-care protocols to support medical oncology and hematology treatments for diseases with and without clinical pathways. We present the types of resources needed to create and build Beacon treatment orders for Epic, the roles of a governance group, and disease teams to oversee Beacon treatment orders and oncology clinical pathways. Given that Elsevier’s ClinicalPath oncology pathways, like McKesson’s Level 1 Pathways by NCCN, are proprietary, we can only present the governance and disease team oversight roles we have identified to oversee the use of these pathways by clinicians and their integration into our Epic EHR. The actual number of Beacon treatment protocols can vary pending individual organization’s decisions to build sequential therapies, such as the common AC-T regimen for breast cancer as an Adriamycin/Cytoxan, the “AC” treatment order to be followed by the paclitaxel “T” treatment order, which would be 2 Beacon treatment protocols, or building it as one AC-T Beacon treatment order. City of Hope initially built components of treatment orders separately but found it more efficient for planning, education, scheduling, preauthorization, and analytics to build standard sequential therapies as one Beacon treatment protocol.

The limitations of our study are that they are limited to Epic Beacon work and the use of Elsevier’s ClinicalPath protocols which cover the majority of diseases treated by oncology organizations. Those few not covered are specified, as well as the number of treatments associated with them. We have not provided the costs to our organization for faculty to participate in leadership and disease pathway work at Elsevier. We have provided the survey from our Epic system for the number of Beacon protocols built on the fly because they were not in our system. We have provided informal surveys of pharmacists and clinicians estimating the time being used to build orders. While a formal survey could add significance to the final numbers of wasted hours, the time is consistent with the number of protocols shown to have been built on the fly and with the hours it takes even experienced Beacon analyst builders to build complete protocols. Survey results are meant to be qualitative and are included in support of the well-established benefits of having comprehensive, complete, centrally built CPOE for oncology safety, efficacy, and efficiencies. The study is limited to data primarily from one large, multi-state academic and community network enterprise, although the informal benchmarking from two other large, multisite academic and community-based cancer programs showed similar time and resource needs and similar numbers of overall oncology treatment orders being used.

Oncology enterprises and programs face many challenges and demands regarding time, staffing, and resources to deliver high-quality cancer care. While oncology pathway programs have been shown to improve outcomes and the value of cancer care and are part of most alternative payment incentive programs by payers, the same is not true for CPOE. Despite extensive evidence showing that computerized provider order entry significantly reduces errors and prevents minor to life-threatening toxicities for vulnerable cancer patients, there are currently no quality metrics by the major value-based payers, ASCO, or other regulatory organizations that specify keeping current with building complex cancer orders in the EHR nor metrics for integrating such orders or patient-specific features into pathway systems to provide more efficient and comprehensive clinical decision support with reportable data. Each organization has been left to implement EHRs, including Epic, focused on the metrics of the state and national regulators, any incentive-based payer contracts, and the interests, expertise, and resourcing of their staff.

Epic, the most extensively used health system EHR in the United States, shares best practices through its Epic Earth and other sites, biannual meetings, and monthly oncology steering board. The oncology steering board’s 12 nationally elected experts advise Epic builders on projects that can better support oncology care. Oncology, however, as only one specialty, and while having had improvements, has yet even to have all the Epic staging forms updated with discrete data entry for standards called out in a December 2017 AJCC editorial and unanimously voted as a priority by the steering board in 2020, which one of the authors, (LB) is a fourth term member [42].

The work to integrate available discrete staging elements from Epic into the ClinicalPath pathways has helped remove clicks and time for entering staging data before navigating pathway options for diseases like breast cancer, where the Epic staging form is robust, and the discrete data pulls into the ClinicalPath system. A limited number of other diseases are fully mapped into ClinicalPath, while some are partially, and most await additional mapping from Epic into the ClinicalPath pathways which will reduce clicks and duplicative data entry to improve efficiencies and pathway navigations.

Linking our clinical trial management system, OnCore, into the ClinicalPath system allows COH’s 800 clinical trials to be loaded by Elsevier into our ClinicalPath disease pathway flow nodes daily. The trial placements are specific to the cancers, biomarkers, and lines of therapy. While challenges remain to remove as many clicks as possible, the ability to show COH sites across the US the sites of each clinical trial, where that clinical trial is active, who the primary investigator is, and a button that alerts the specific clinical trial team to evaluate a patient for a trial improves the value of pathway navigation for clinicians. Additional clinical trial information is available from the pathway tool with a link to clinicaltrials.gov. Further improvements are expected as Dr. Kim at COH has developed a clinical trial tool that can be used as a standalone and as a potential link within our ClinicalPath pathway tool to rapidly let clinicians review and download the eligibility, inclusion/exclusion criteria and the COH consent form as well as other COH specific trial information.

## 5. Conclusions

Oncology program leaders need a framework of components to understand and resource oncology clinical pathways and CPOE as the major components of CDSS for oncology. This framework to support CDSS and the components for clinical pathways and CPOE can be used with data from individual enterprises to support business cases for the building, updating, maintaining, and regulatory oversight of Beacon treatment orders in Epic as well as for orders in other electronic medical records. Beacon orders are essential CPOE for Epic practices, which is a key component of Clinical Decision Support Systems along with oncology clinical pathways. We share this work and the findings of our Pathways and Protocols Program team in developing an enterprise-wide program and establishing the business case for CPOE resources to build and maintain Beacon treatment orders in the hopes that others can leapfrog programs and resource support at their institutions. We hope others can program and build business cases to staff their organizations to keep Epic Beacon or other HER systems’ oncology treatment orders up to date, linked to comprehensive oncology clinical pathways with clinical trials and analytics to improve safety and efficiencies for oncology patients and staff.

## Figures and Tables

**Table 1:** Survey results of prioritization of ClinicalPath associated Beacon protocol builds.

Priority by Usage Frequency	Number of Regimens (n = 326)
1-Use once every 1–4 weeks	36
2-Use once every 1–3 months	75
3-Use once every 4–12 months	89
4-Not currently or rarely used	126

**Table 2:** Estimated monthly staff time to build or modify unbuilt Beacon orders based on Epic study.

Role	% Time Editing Beacon Orders	Estimated Hours/Month Editing Beacon Orders	Estimated Hours/Year Editing Beacon Orders
MD	32%	30.8–50.8	369–610
PharmD	37%	35.6–58.8	427–705
APP	19%	18.3–30.2	219–362
RN	3%	2.9–4.8	35–57
Others	9%	8.7–14.3	104–172
Total Hours Used:		96–159	1154–1906

Hours based on our Epic study of 576 providers who modified or built new Epic orders from April 2022 through April 2024. Data are primarily from California sites, as CAP sites were only on Epic for 6 months of the study. (October 2023 through April 2024). Based on 7500 studies annually needing to be built new or from modifying an order. Time ranges from 5 to 40 minutes based on complexity.

**Table 3:** Time used by community site clinicians to build Beacon protocols that could be used to see new patients.

Sites	# Hematologists	# Medical Oncologists	MD Time per Day on Orders	PharmD Help	Total Time per Month per MD	New Patient Potential per Month per Site
CA Network	48		0.25 h	No	6 h	72 h
CAP-Chicago	1	8	1.5 h	Yes	28 h	63 h
CAP-Atlanta	2	6	1 h	No	20 h	40 h
CAP-Phoenix	12		1.25 h	No	25 h	75 h

Time per regional community site to build Beacon orders using 5 days of clinic per week per doctor. Total time per month is calculated by the time per doctor per month times the number of doctors at each site. New patient potential is calculated from doctors being able to see at least one new patient for every 4 h freed from order development. # = number of hematologists or number of medical oncologists.

**Table 4:** Clinical pharmacist time per site being used to build Beacon orders with commentary from clinical pharmacists.

Sites with PharmDs	# of PharmDs	Time Spent per PharmD on Beacon Orders	Comments
Duarte (outpatient only)	17	1–1.5 h/PharmD	Could free 17–25 h of PharmD time per day
Lennar in Irvine	1–2	1.5–2 h/PharmD	Could free 3–4 h per PharmD per day
Chicago	15	1 h/PharmD	Could free 15 h of PharmD time per day based on 50 patients/day
Phoenix	3–4	0.25 h/PharmD per patient	It can take 30 min per Beacon order for complicated protocols

PharmD = clinical pharmacists. # = number of PharmDs.

**Table 6:** Recommended staffing to catch up and maintain COH Epic Beacon protocols.

	FY 25 and FY 26 Q1	FY27 Forward
Beacon Protocol Catch-Up Project		
Informatics Pharmacists	2 FTE (1 have + 1 new)	N/A
Operations Project Manager	0.5 FTE new	N/A
Epic Beacon Systems Analyst (builder)	3 FTE new contractor	N/A
Epic Willow Systems Analyst (builder)	1 FTE existing	N/A
IT Project Manager	0.20 FTE contractor	N/A
Annual Beacon Protocol Maintenance		
Informatics Pharmacist	1 FTE new	3 FTE
Operations Project Manager	0.5 FTE new	0.5 FTE
Epic Beacon Systems Analyst (builder)	2 FTE existing	2 FTE
Epic Willow Systems Analyst (builder)	1 FTE existing	1 FTE
IT Project Manager	N/A	

FTE means full-time equivalent—new means new hires or to be contracted if noted as a new contractor. FY = fiscal year. Q1 = quarter one. N/A = not available as catch-up is projected to be done.

**Table 7:** Comparison of Beacon protocol resources currently and proposed for COH and two other multisite, multistate cancer programs using Epic.

		COH Current Orders and Staffing	Other NCI Cancer Center Orders and Staffing	Large Integrated Multistate Health System	NEW COH Orders and Staffing Needs
Active Protocols (total)		3100	~5000	2400	3500
Standard of Care (SOC)		1100	~1800	2400	1500
	Beacon Analyst/builder	1.5	2.4–3.2	3	4.5 (+3)
	Informatics PharmD	1	4–5.2	5	3 (+2)
	Medication/Willow builder	2	3–4	1	3 (+1)
Clinical Trial Protocols (IRB)		2000	~3000	N/I	2000
	Beacon Analyst/builder	7.8	4.8–5.6	N/I	No change
	Informatics Pharm/RNs	14	7.8–9	N/I	No change
	Medication/Willow builder	2	6–7	----	No change

COH: Clinical Trial Protocols at COH built by 14 separate research PharmDs. Beacon: 13 analysts. In total, 10% for SOC builds, and 60% for IRB = investigational review board builds. Willow: 4 out of 8 analysts are medication builders, 2 are dedicated to SOC builds, and 2 for IRB. Other NCI = national cancer institute Cancer Center: of 14 Beacon Analysts, 6 are informatic pharmacists. Of 17 Willow Analysts, 7 are informatic pharmacists. Content and build work is disease-specific; an estimated 0.3–0.4 FTE per FTE is dedicated to SOC work, and 0.6–0.7 FTE per FTE are for IRB works. RN = registered nurse. N/I = no information available.

**Table 8:** Standardized Beacon protocol order components for COH enterprise as applicable.

Treatment Schedule	Treatment Details	Patient Instructions	Nursing Instructions	Supportive Care	Regulatory Requirements
Financial Authorization	Drugs, dosing, and scheduling	Education on regimen and home care	Infusion and administration instructions	Neutropenic fever	Oral chemo compliance check
Lab and Imaging orders	Dose modification and hold rules	Lab, imaging, and visit schedule	Treatment details-timing, sequencing, mixing	Infection prevention and prophylaxis	REMS program
Treatment days	Antiemetic and hypersensitivity orders	Treatment schedule	Treatment parameters for dosing or not	VTE prevention	Hepatitis B/C and TB screening
MD and other visits	Specialty Pharmacy use	Fertility preservation info/referrals	Emergency medications and extravasations	TLS prevention	Pregnancy screening

Lab = laboratory orders, VTE = venous thromboembolism, TLS = tumor lysis syndrome, REMS = Risk Evaluation and Mitigation Strategy, TB = tuberculosis.

**Table 9:** Standardized naming and components for Beacon protocols.

Standardized Beacon Protocol Format
Protocol name
Protocol description
Emetogenicity designation
Reference to treatment or landmark trials
Display template of orders

**Table 10:** Drugs by category and type to be included in the four risk categories of antiemetics.

Drug Class	Medications	HEC	MEC	LEC	MIN
NK1 RA	Outpatient: aprepitant Inpatient fosaprepitant	**X**			
5HT3 RA	IV dominant: palonosetron Oral Dominant: ondansetron	**X**	**X**	IV or Oral ondansetron	PRN ondansetron
Steroid	dexamethasone *	X4 days	X3 days		
5H2, 5H3, dopamine, D2 RA	olanzapine **	PRN X4 days	PRN X3 days		

IV or oral dominant premedications to be built for every protocol for physician choice with these standard medications by emetogenic risk category consistent with NCCN guidelines. * Steroids will be avoided in patients receiving post-transplant cyclophosphamide for GVHD prophylaxis. ** Olanzapine will be avoided in transplant patients. Four-day course for high emetogenic regimens and a three-day course for medium emetogenic regimens. HEC = highly emetogenic chemotherapy, MEC = moderate emetogenic chemotherapy, LEC = low emetogenic chemotherapy, MIN = minimal emetogenic chemotherapy. PRN = only as needed, IV = intravenous, NK1 RA = neurokinin 1 receptor antagonist. 5HT3 RA = 5-hydroxytryptamine 3 receptor antagonist. 5H2 = histamine H2 receptor antagonists, 5H3 = same as 5HT3 RA, D2 RA = dopamine D2 receptor antagonist.

**Table 11:** Antiemetic regimen types, including hypersensitivity and medication alterations for protocols.

Drug	HEC	MEC	LEC	MIN
	HEC multiday			
	HEC	MEC	LEC	MIN
	HEC no NK1 RA			
	HEC min steroid	MIN-min steroid		
Paclitaxel q2–3 week	HEC-Paclitaxel	MEC-Paclitaxel	LEC-Paclitaxel	
	HEC no NK1 RA-Paclitaxel			
Paclitaxel weekly	HEC-Paclitaxel weekly	MEC-Paclitaxel weekly	LEC-Paclitaxel weekly	
	HEC no NK1-Paclitaxel weekly			
Docetaxel	HEC-Docetaxel	MEC-Docetaxel	LEC-Docetaxel	
	HEC-no NK1 RA-Docetaxel			
Pemetrexed	HEC-Pemetrexed	MEC-Pemetrexed	LEC-Pemetrexed	
	HEC no NK1 RA-Pemetrexed			

Standard premedication groupers for the 24 medication combinations to address different antiemetic risks, different days of antiemetic risks, those where NK1 RA = neurokinin 1 receptor antagonist, needs to be removed, or steroids need to be minimized or adjusted for hypersensitivity and antiemetic treatment coordination. HEC—highly emetogenic chemotherapy, MEC—moderate emetogenic chemotherapy, LEC—low emetogenic chemotherapy, MIN—minimal emetogenic chemotherapy, min-steroid—minimize steroid, oral only needed.

## Data Availability

Original data sets from which the graphs and tables were made are not publicly available. They are maintained by L.D.B., Y.H., and B.M. who can be contacted with any questions about the data or its presentation.
